# Supplementary material for: Selective Delivery of Tofacitinib Citrate to Hair Follicles Using Lipid-Coated Calcium Carbonate Nanocarrier Controls Chemotherapy-Induced Alopecia Areata
Source: Int J Mol Sci. 2023 May 8;24(9):8427. doi: 10.3390/ijms24098427 (PMC10179728; doi:10.3390/ijms24098427)
Supplement: Supplementary file 1 [file ijms-24-08427-s001.zip › ijms-2279263-supplementary.pdf]

Supporting Information

S1 Materials

Dulbeccos Modified Eagles Medium (DMEM) and 0.25% trypsin EDTA were purchased from Gibco (Grand Island, NY, USA). Penicillin Streptomycin Solution（P/S）was purchased from Beijing shenghang Biotechnology Co., Ltd. (Beijing, China). William's E medium was purchased from Wuhan Yipu Biotechnology Co. (Hubei, China). Fetal bovine serum (FBS) was purchased from Zhejiang Tianhang Biotechnology Co., Ltd. (Zhejiang, China). Phenobarbital sodium was provided by Beijing Huayehuanyu Chemical Co., Ltd (Beijing, China). TritonX-100 and 3-(4,5-Dimethylthiazol-2-yl)-2,5-diphenyltetrazolium bromide (MTT) were purchased from Wuhan Kerui Biotechnology Co., Ltd (Wuhan, China). Bovine serum albumin (BSA) was purchased from Yancheng Saibao Biotechnology Co., Ltd (Jiangsu, China). Neutral resin was purchased from Beijing pulilai Gene Technology Co., Ltd (Beijing, China). Mayer hematoxylin staining solution was purchased from Beijing leagene Biotechnology Co., Ltd.（Beijing，China）. Collagenase IV and DNase I were purchased from Gibco (Grand Island, NY, USA). Red blood cell lysate and eosin solution were purchased from Beijing solarbio Technology Co., Ltd. (Beijing, China). Goat serum was purchased from Beijing biosynthesis Biotechnology Co., Ltd. (Beijing, China). Mounting Medium with DAPI was purchased from Abcam (Cambridge, MA, USA). Interferon-γ (IFN-γ) was purchased from Tonglihaiyuan Biotechnology Co., Ltd. (Beijing China).

S2 Drug loading (DL%) and encapsulation efficiency (EE%)

PL/ACC-TFC NPs solution (20 μL) was added to the mixture solution (980 μL, methanol: DMSO=2:1, V: V), then the supernatant was removed by centrifugation at 10,000 rpm for 10 min after sonicated for 30 min. The drug loading (DL%) and encapsulation efficiency (EE%) of TFC in PL/ACC-TFC NPs was detected under the guidance of TFC standard curve using High-Performance Liquid Chromatography (HPLC) analysis with an Elite Hypersil ODS2 column (5 μm, 4.6×250 mm). The mobile phase was 10 mM ammonium acetate in water (pH=5 adjusted with acetic acid) and methanol (55:45, v/v) at 30 °C. The flow rate and injection volume were 1.0 mL min^{-1}and 100 μL, respectively, and the detection wavelength was 287 nm. The drug loading amount (DL %) and encapsulation efficiency (EE %) were followed the formulas.

$$Drug\ Loading(DL\%) = \left(\frac{\text{Weight of TFC in } PL/ACC-TFC}{\text{Weight of PL/ACC−TFC}}\right) \times 100\%$$

$$Encapsulation\ efficiency\ (EE\%) = \left(\frac{\text{Weight of TFC in PL/ACC−TFC}}{Weight\ of\ TFC\ invested}\right) \times 100\%$$

S3 The cumulative permeation amount per unit area and Steady-state transdermal rate per unit area

The cumulative permeability per unit area was calculated as follow[2].

$$Q = (V \times Cn + \sum_{i=1}^{i=n-1} Ci)/A$$

In the above formula: V was the volume of the receiving cell, C_n was the drug concentration measured by sampling at the nth point, and A was the effective permeation area of the diffusion cell.

The steady-state transdermal rate per unit area was the slope of the cumulative drug penetration per unit area versus the linear part of the time plot.

S4 Immunohistochemical staining

Immunohistochemical staining of human hair follicle organs: Hair follicle organ was collected and prepare frozen section. Frozen section was permeabilized with 0.5% Triton X-100 for 10 min, blocked with 1% BSA and 10% goat serum for 60 min at room temperature and then incubated with mouse anti human HLA-A, B, C (MHC-Ⅰ) primary antibody (Leinco, W6/32, 1:100) overnight at 4 °C. After washing with PBS for three times, the sections were incubated with Dylight 598-conjugated secondary antibody for 1 h at room temperature in the dark followed by staining with DAPI. The sections were imaged with a fluorescence microscope.

Immunohistochemical staining of mouse skin tissue: The skin tissues from CYP-induced alopecia areata mice were collected and prepare frozen section. Sections were first circled with an immunohistochemical oil pen, blocked with 10% goat serum and 1% BSA in PBS, permeabilized with 0.5% Triton X-100 in PBS for 10 min and incubated with APC-anti mouse I-A/I-E (MHC-Ⅱ) antibody (Biolegend, 1:100) overnight at 4°C. Mounting medium with DAPI was used as a nuclear counterstain.

Supplementary Tables

Table S1. Antibodies used in the study.

Antibodies	Company	Catalog	Application
Anti-Human HLA-A, B, C (MHC Class I)	Leinco	H263	IF
Goat Anti-Mouse (Dylight 594 -conjugated)	yakeyin	A23410	IF
Anti-MHC Class II (APC-conjugated)	Biolegend	107614	flow cyt
Anti-CD8a (APC-conjugated)	Biolegend	100711	flow cyt
Anti-CD314(NKG2D) (FITC-conjugated)	Biolegend	115711	flow cyt

Table S2. Formulation of ACC NPs and PL/ACC-TFC NPs.

Sample code	Amount of $CaCl_2$ (g)	Concentration of water in ethanol (v/v)	Amount of NH_4HCO_3 (g)	Particle size of ACC NPs (nm)	Particle size of PL/ACC-TFC NPs (nm)
1	0.1	0.1%	2.5	107.8±4.7	155.2±9.3
2	0.2	0.2%	2.5	200.6±14.7	285.1±8.0
3	0.2	0.4%	2.5	403.1±10.4	474.8±16.9

Supplementary figures

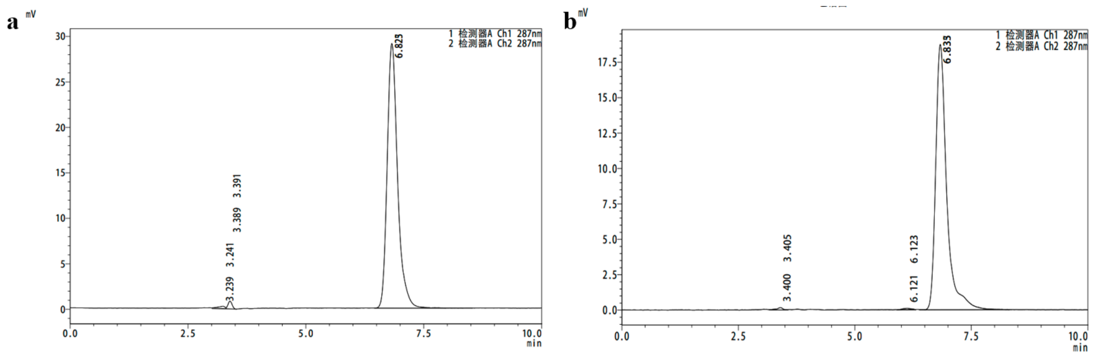

Figure S1. High performance liquid chromatography (HPLC) chromatogram：（a）free TFC solution，（b）PL/ACC-TFC NPs

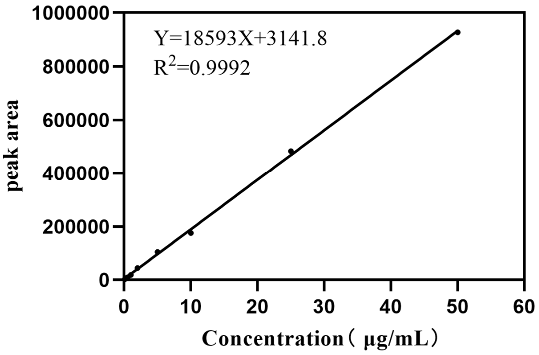

Figure S2. Standard curve of TFC

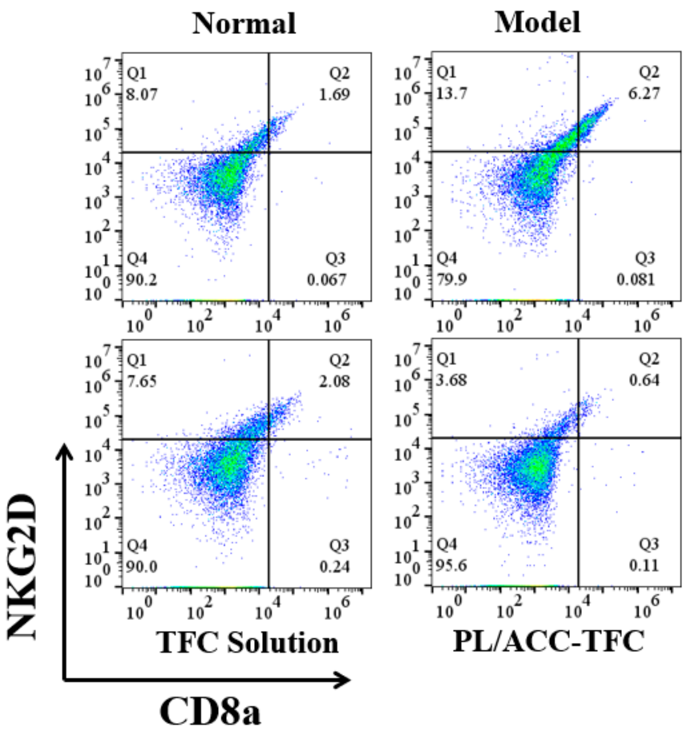

Figure S3. Flow cytometry analysis of NKG2D+ CD8+ T cell levels.

References

[1] Zhu H, Liu Q, Miao L, Musetti S, Huo M and Huang L 2020 Remodeling the fibrotic tumor microenvironment of desmoplastic melanoma to facilitate vaccine immunotherapy *Nanoscale* **12** 3400-10

[2] Shi T, Lv Y, Huang W, Fang Z, Qi J, Chen Z, Zhao W, Wu W and Lu Y 2020 Enhanced transdermal delivery of curcumin nanosuspensions: A mechanistic study based on co-localization of particle and drug signals *International Journal of Pharmaceutics* **588**
